# Serum miR-375-3p increase in mice exposed to a high dose of ionizing radiation

**DOI:** 10.1038/s41598-018-19763-7

**Published:** 2018-01-22

**Authors:** Mitsuru Chiba, Satoru Monzen, Chihiro Iwaya, Yuri Kashiwagi, Sunao Yamada, Yoichiro Hosokawa, Yasushi Mariya, Toshiya Nakamura, Andrzej Wojcik

**Affiliations:** 1 Department of Bioscience and Laboratory Medicine, Graduate School of Health Sciences, Hirosaki University, 66−1, Hon-cho, Hirosaki, Aomori, 036-8564 Japan; 2 Department of Radiation Sciences, Graduate School of Health Sciences, Hirosaki University, 66−1, Hon-cho, Hirosaki, Aomori, 036-8564 Japan; 3 Department of Medical Technology, Hirosaki University School of Health Sciences, 66−1, Hon-cho, Hirosaki, Aomori, 036-8564 Japan; 4 Department of Radiology and Radiation Oncology, Mutsu General Hospital, 1−2−8, Kogawa-machi, Mutsu, Aomori, 035-0071 Japan; 5 Department of Molecular Biosciences, The Wenner Gren Instititute, Stockholm University, Svante Arrhenius väg 20 C, 10691 Stockholm, Sweden; 6 Department of Radiobiology and Immunology, Institute of Biology, Jan Kochanowski University, ul. Swietokrzyska 15, 25-406 Kielce, Poland

## Abstract

Exposure to high-doses of ionizing radiation (IR) leads to development of a strong acute radiation syndrome (ARS) in mammals. ARS manifests after a latency period and it is important to develop fast prognostic biomarkers for its early detection and assessment. Analysis of chromosomal aberrations in peripheral blood lymphocytes is the gold standard of biological dosimetry, but it fails after high doses of IR. Therefore, it is important to establish novel biomarkers of exposure that are fast and reliable also in the high dose range. Here, we investigated the applicability of miRNA levels in mouse serum. We found significantly increased levels of miR-375-3p following whole body exposure to 7 Gy of X-rays. In addition, we analyzed their levels in various organs of control mice and found them to be especially abundant in the pancreas and the intestine. Following a dose of 7 Gy, extensive cell death occurred in these tissues and this correlated negatively with the levels of miR-375-3p in the organs. We conclude that high expressing tissues of miR-375-3p may secrete this miRNA in serum following exposure to 7 Gy. Therefore, elevated miR-375-3p in serum may be a predictor of tissue damage induced by exposure to a high radiation dose.

## Introduction

A severe consequence of exposure to high doses of ionizing radiation (IR), whether in consequence of an accident or a deliberate terrorist attach with the use of radiation sources, is the acute radiation syndrome (ARS) involving bone marrow and gastrointestinal necrosis and often leading to multi organ disease syndrome (MODS). The treatment and possible prevention of ARS requires that the dose absorbed by the victim is known^1^. ARS can fully develop after 21–60 days post exposure, with earlier symptoms occurring during the prodromal phase (<48 h after exposure) and the latent phase (2–20 days after exposure)^1,2^. ARS can be treated by antibiotics, cytokines, blood transfusion and/or stem cell transplantation which prevent infections, excessive immune response and tissue break down, but the treatment success is inversely correlated with the time between radiation exposure and its onset^3–8^.

The gold standard of biological dosimetry is the chromosomal aberration assay in peripheral blood lymphocytes which, following high doses of radiation, can be modified by premature chromosome condensation (PCC)^9,10^. The doses received by the three severely exposed victims of the Tokai-mura criticality accident in 1999 (JCO accident, Japan) were analyzed by PCC, but this was only thanks to the possibility of blood collection within hours after irradiation, before the occurrence of leukopenia^9^. The disadvantage of the chromosomal aberration assay is that it is time consuming due to the necessity to culture lymphocytes^11,12^. In case of a large radiation emergency this is a serious problem and there is a need to develop new, faster assays which allow for a quick triage of people who are at risk of developing ARS^13,14^.

Recently, several studies indicated that stable miRNAs in blood serum/plasma may be suitable as biomarkers of exposure^15,16^. MiRNAs are endogenous, 18–25 nucleotides non-coding RNAs, which were discovered in 1993 in *Caenorhabditis elegans*^17^. MiRNA genes are transcribed by RNA polymerase II into long primary miRNAs (pri-miRNAs). These pri-miRNAs are processed in the nucleus into 70–80 nucleotide precursor miRNAs (pre-miRNAs) by the RNase III enzyme Drosha. Pre-miRNAs are then actively transported from the nucleus to the cytoplasm by Exportin-5 where they are processed by the enzyme Dicer. Dicer is an RNase III endonuclease that cleaves the pre-miRNAs into their mature forms which become stably associated with RNA-induced silenced complexes^18^. MiRNAs regulate gene expression post-transcriptionally both in animals and plants. Recently, a number of miRNAs were found in extracellular spaces, and some of these were embedded in extracellular vesicles (EVs) such as exosomes or apoptotic bodies. Valadi *et al*. discovered that exosomes including various mRNAs and miRNAs were released from cells to extracellular space. These extracellular RNAs can be taken up by other cells^19^. In addition, complexes of extracellular miRNAs and Argonaute 2, a high-density lipoprotein, as well as other RNA-binding proteins were detected in body fluids and cell culture supernatants^20–23^.

The diagnostic value of miRNAs circulating in body fluids was recently suggested. Nonaka *et al*. reported that miR-103 and miR-720 in serum serve as biomarkers for patients with colorectal cancer^24^. Likewise, Jacob *et al*. showed that the expression of miR-150, miR-200b, and miR-762 in blood serum of mice changes in relation to the dose of radiation^25^.

An intriguing question remains about the origin and role of these miRNAs. Which organs or tissues are they released from and why? In the present study we tried to find an answer to these questions by analyzing the levels of miRNA in serum of mice which received whole body doses of X-rays. The ultimate aim was to identify and validate miRNA which could serve as a fast and reliable biodosimeter.

## Results

### Exposure of mice to 7 Gy induces a strong ARS

**Figure 1:**
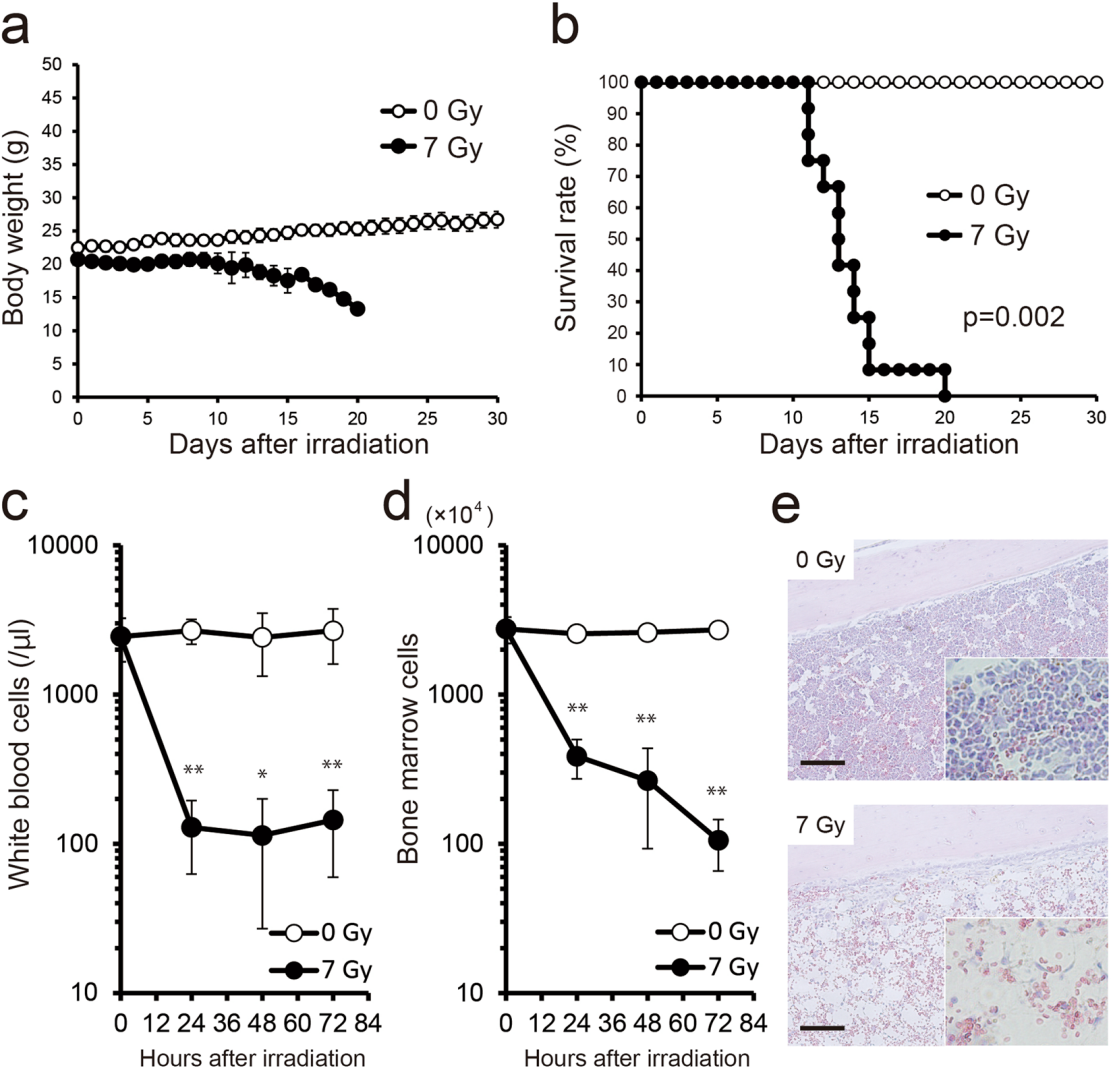
Effects of exposure to 7 Gy of X-ray. (**a**) Daily body weight changes of irradiated C57BL/6 N male mice (7 Gy, *n* = 12) and non-irradiated controls (0 Gy, *n* = 6) until day 30 after irradiation. The data are expressed as means ± standard deviations. (**b**) A Kaplan-Meier plot for the survival of irradiated and control mice. Statistical difference between irradiated animals and controls was determined by log-rank test, with *P* ≤ 0.05 considered as significant. (**c**) Mean counts of peripheral white blood cells (PWBC) in irradiated mice. (**d**) Counts of bone marrow cells in irradiated mice. PWBC and bone marrow cells decreased significantly at 24 h, 48 h and 72 h after irradiation compared to 0 Gy (each *n* = 6−7). Statistical significance was examined by Student’s *t*-test (*P ≤ 0.01, **P ≤ 0.001). (**e**) Hematoxylin and eosin staining of decalcified thighbone sections in irradiated mice. HE staining was performed to visualize the fine structures of the thighbone. Bone marrow cells in mice at 72 h after 7 Gy irradiation decreased clearly as compared to 0 Gy. Scale bars, 100 μm.

It is known that a whole body dose of 7 Gy X-rays is lethal to mice, involving bone marrow depletion and reduction of peripheral white blood cells (PWBC)^26^. In order to confirm these results and verify that a dose of 7 Gy induces a strong ARS in our mouse model, we monitored the changes of body weight and the level of animal survival over a period of 30 days. Body weight gradually increased in non-irradiated mice (0 Gy), whereas it decreased in irradiated animals, starting from day 10 *post radiationem* (p.r.) (Fig. 1a). Survival rate was visualized by a Kaplan-Meier plot (Fig. 1b). In the non-irradiated group it was 100% on day 30, whereas in the irradiated group it gradually decreased from day 12 p.r. and reached 0% on day 20. The median survival time of the irradiated group was 13 days. The difference between the irradiated and non-irradiated group was highly significant (*P = *0.002).

We also analyzed the counts of PWBC and bone marrow cells using a counting chamber. Compared to the non-irradiated group (0 Gy), counts of PWBC and bone marrow cells decreased significantly after 24 h, 48 h and 72 h p.r. (Fig. 1c and d). A severe decrease in bone marrow cell numbers was also evident from histological images of thigh bones at 72 h p.r. (Fig. 1e). These results demonstrate that a whole body dose of 7 Gy X-rays leads to the formation of a strong ARS in our mice.

### MiRNA expression changes in mouse serum after exposure to 7 Gy of X-rays

**Figure 2:**
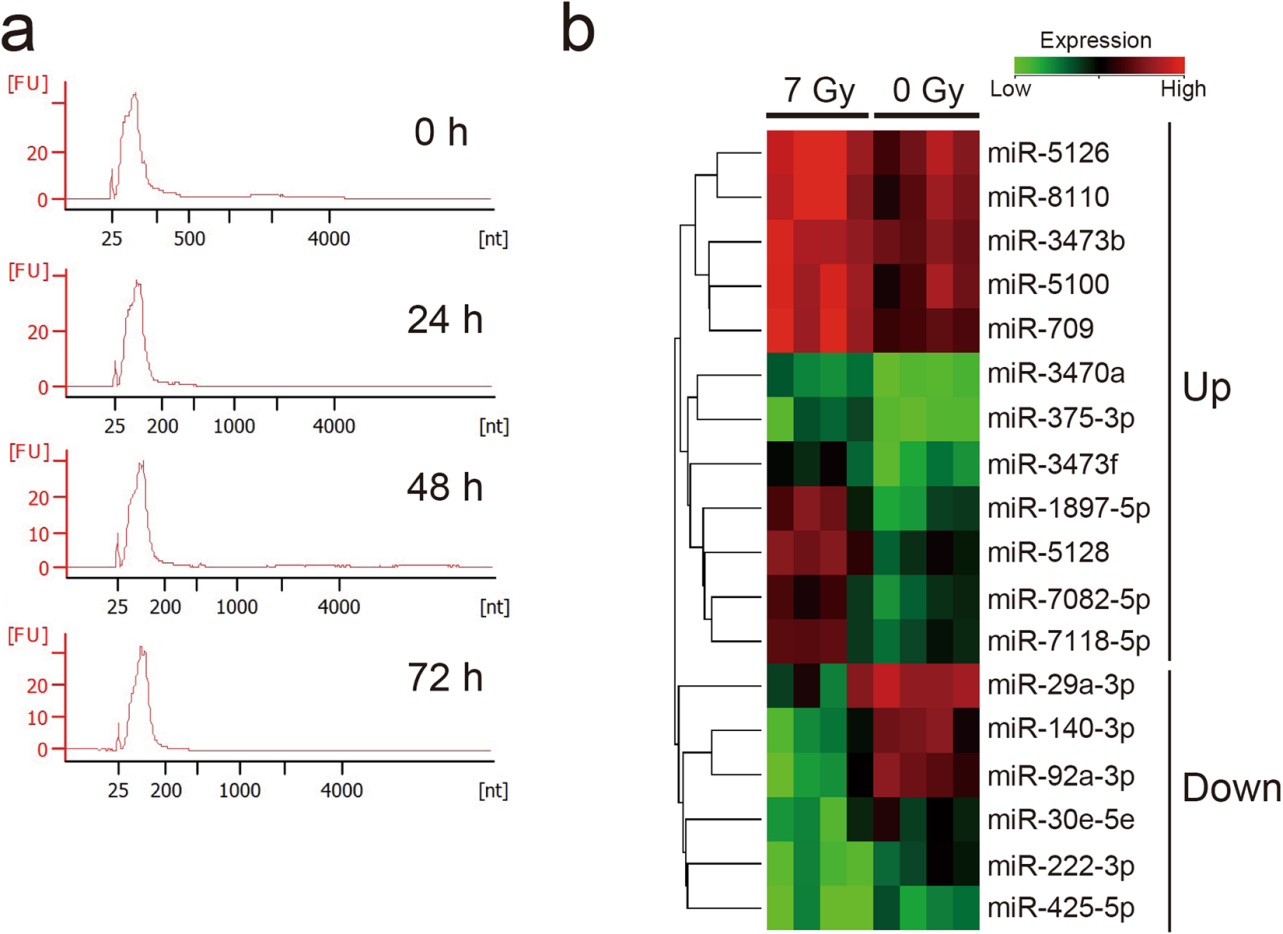
Identification of differentially expressing miRNAs in serum of mice exposed to 7 Gy of X-rays. (**a**) Detection of serum RNAs in mice. Sera were obtained from mice at 0 h, 24 h, 48 h and 72 h after 7 Gy irradiation. Serum RNAs were extracted using Isogen II reagent (Nippongene) as described in materials and methods. Extracellular small RNAs were detected in all samples using an Agilent Bioanalyzer (Agilent Technologies). (**b**) Expression analysis of increasing and/or decreasing miRNAs in serum of mice (each *n* = 4) exposed to a 7 Gy of X-ray using an Agilent mouse miRNA microarray. Heat map showing variations in a part of 18 radio-responsive miRNAs, identified by the Student’s *t*-test with a cut off *P*-value of 0.05 and 2.0-fold changes using GeneSpring GX14.5 software (Agilent Technologies). Increasing 12 miRNAs and decreasing 6 miRNAs were identified, respectively.

At 0 h, 24 h, 48 h and 72 h after 7 Gy irradiation, serum RNAs in blood sera were extracted using Isogen II reagent as described in materials and methods. Peaks of 25–200 nucleotides were detected by an Aglient 2100 Bioanalyzer and pico kits, and 18 S and 28 S ribosomal RNAs were not detected (Fig. 2a). This indicated a high level of extracellular small RNAs in sera. To identify increasing and/or decreasing miRNAs in sera of irradiated mice, we performed the Agilent mouse miRNA microarray assay. Cyanine-3 (Cy3) labelled miRNA was synthesized using the miRNA complete labelling and hyb kit (Agilent Technologies, Santa Clara, CA, USA) and 30 ng of total RNA isolated from 200 µl of serum as described in materials and methods. The levels of miRNAs in 7 Gy irradiated samples (72 h) were compared to non-irradiated samples (0 Gy). Increasing 12 and decreasing 6 miRNAs were identified by Student’s *t*-tests with a cut off *P*-value of 0.05 and 1.5-fold changes (Fig. 2b and Supplementary Table S1).

### MiR-375-3p and miR-709 levels increase in serum of mice exposed to 7 Gy of X-ray

**Figure 3:**
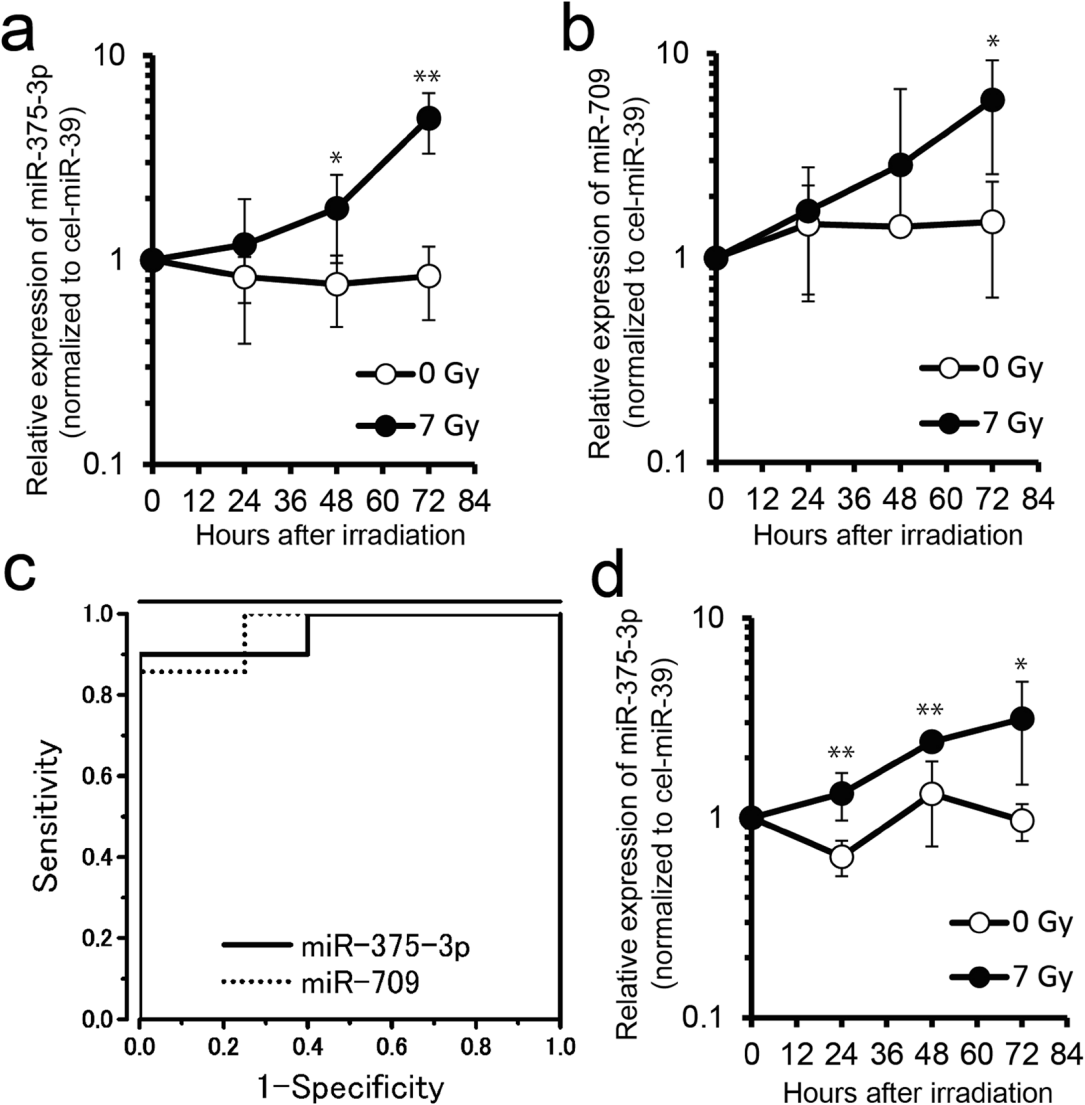
MiR-375-3p and miR-709 expression in serum of mice exposed to 7 Gy of X-ray. (**a** and **b**) Total serum RNAs were extracted from 50 μl aliquots. The expression of (**a**) miR-375-3p and (**b**) miR-709 in serum of mice at 0 h, 24 h, 48 h and 72 h after 7 Gy X-irradiation (each *n* = 4−7), measured by reverse transcription quantitative polymerase chain reaction (RT-qPCR). (**c**) Receiver-operating characteristic (ROC) analysis of miR-375-3p and miR-709 at 72 h after irradiation between 0 Gy and 7 Gy (each *n* = 7−10). (**d**) The expressions of miR-375-3p in extracellular vesicles (EVs) derived from serum of 7 Gy irradiated mice (each *n* = 5−7). EVs were collected using ExoQuick reagent (System Biosciences). Cel-miR-39 was used as external control. Expression levels of miR-375-3p and miR-709 were determined by RT-qPCR using the comparative Ct methods. Statistical significance was examined by Student’s *t*-test (*P ≤ 0.05, ** *P* ≤ 0.01 vs 0 Gy).

Selected radio-responsive miRNAs identified from the cluster analysis shown in Fig. 2b were further validated by reverse transcription quantitative polymerase chain reaction (RT-qPCR) as described in materials and methods. We chose miR-375-3p and miR-709 as candidate serum biomarkers of a strong ARS because these miRNAs were most frequently reported among increasing 12 miRNAs. At 48 h and 72 h after 7 Gy irradiation the expression of miR-375-3p in serum was significantly increased as compared with 0 Gy: respectively, 2.35-fold (*P* ≤ 0.05) and 5.91-fold (*P* ≤ 0.01) (Fig. 3a). At 72 h after 7 Gy irradiation the expression of miR-709 in serum was significantly increased as compared with 0 Gy: 3.94-fold (*P* ≤ 0.05) (Fig. 3b). We carried out a receiver-operating characteristic (ROC) analysis, and found the area under the curve (AUC) of miR-375-3p and miR-709 to be 0.960 and 0.964, respectively (Fig. 3c). These values suggest that miR-375-3p and miR-709 in serum are sensitive and specific biomarkers of exposure to 7 Gy.

In order to investigate whether serum miR-375-3p is expressed inside EVs, we isolated RNAs from EV pellets collected using an ExoQuick Solution (System Biosciences, San Francisco, CA, USA) and then performed RT-qPCR. The expression of miR-375-3p in serum was significantly increased at 24 h, 48 h and 72 h after 7 Gy irradiation as compared with 0 Gy: respectively, 2.07-fold (*P* ≤ 0.01), 1.82-fold (*P* ≤ 0.01) and 3.23-fold (*P* ≤ 0.05) (Fig. 3d). This result suggests that EVs are the origin of increased serum miR-375-3p levels in irradiated mice.

### MiR-375-3p and miR-709 are highly expressed in pancreas of control mice

**Figure 4:**
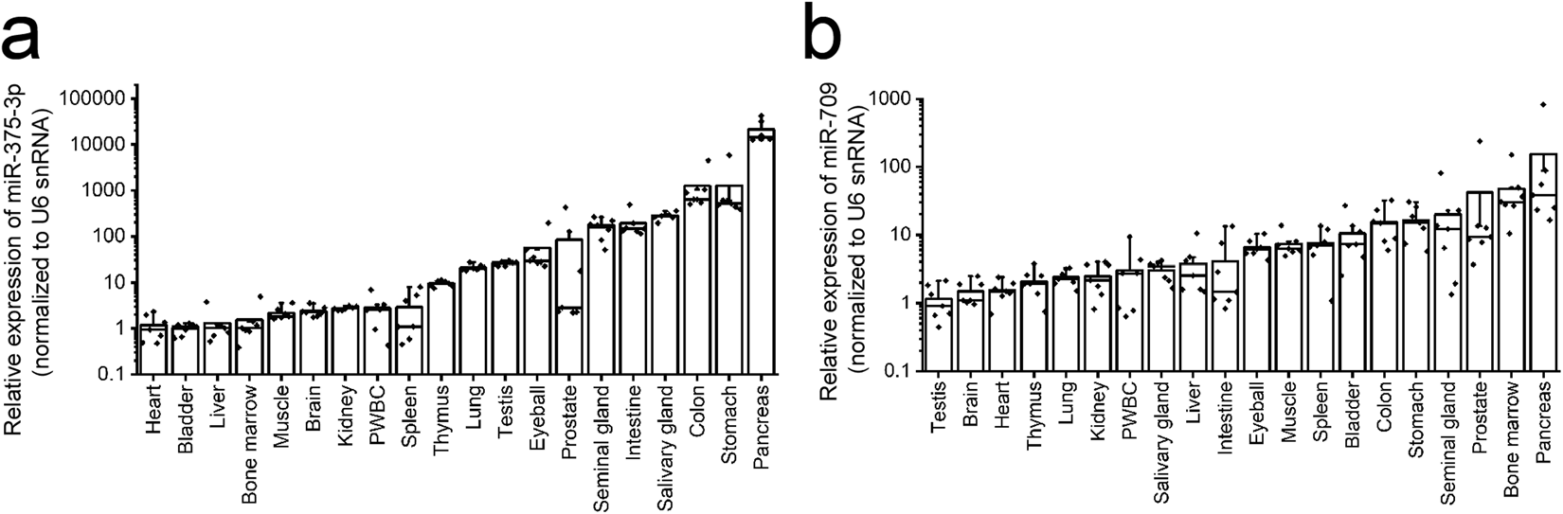
High expression of miR-375-3p and miR-709 in pancreas. (**a** and **b**) The expression of (**a**) miR-375-3p and (**b**) miR-709 in organs/cells of control mice (each *n* = 6−7). Total RNAs from 2 cell systems [peripheral white blood cells (PWBC) and bone marrow cells] and 18 organs (brain, eyeball, salivary gland, thymus, heart, liver, stomach, pancreas, kidney, spleen, lung, testis, prostate, bladder, seminal gland, intestine, colon and muscle) were extracted using Isogen II reagent (Nippongene) for RT-qPCR. Expression values of miR-375-3p and miR-709 were determined by the comparative Ct method and normalized using the values of heart and testis set at 1.0, respectively. U6 small nuclear RNA (snRNA) was used as an internal control.

In order to identify the possible origin of the radiation-induced miR-375-3p and miR-709 in sera of mice, we analyzed the expressions of miR-375-3p and miR-709 by RT-qPCR in various cells and organs of the control animals. Total RNAs from PWBC and bone marrow cells as well as from 18 organs (brain, eyeball, salivary gland, thymus, heart, liver, stomach, pancreas, kidney, spleen, lung, testis, prostate, bladder, seminal gland, intestine, colon and muscle) were extracted using the Isogen II reagent for RT-qPCR. MiR-375-3p and miR-709 was highly expressed in pancreas (Fig. 4a and b). This suggests that the high, radiation-induced levels of miR-375-3p and miR-709 in serum may be released from this organ.

### Radiation-induced cell death triggers decrease of miR-375-3p expression in pancreas and intestine

**Figure 5:**
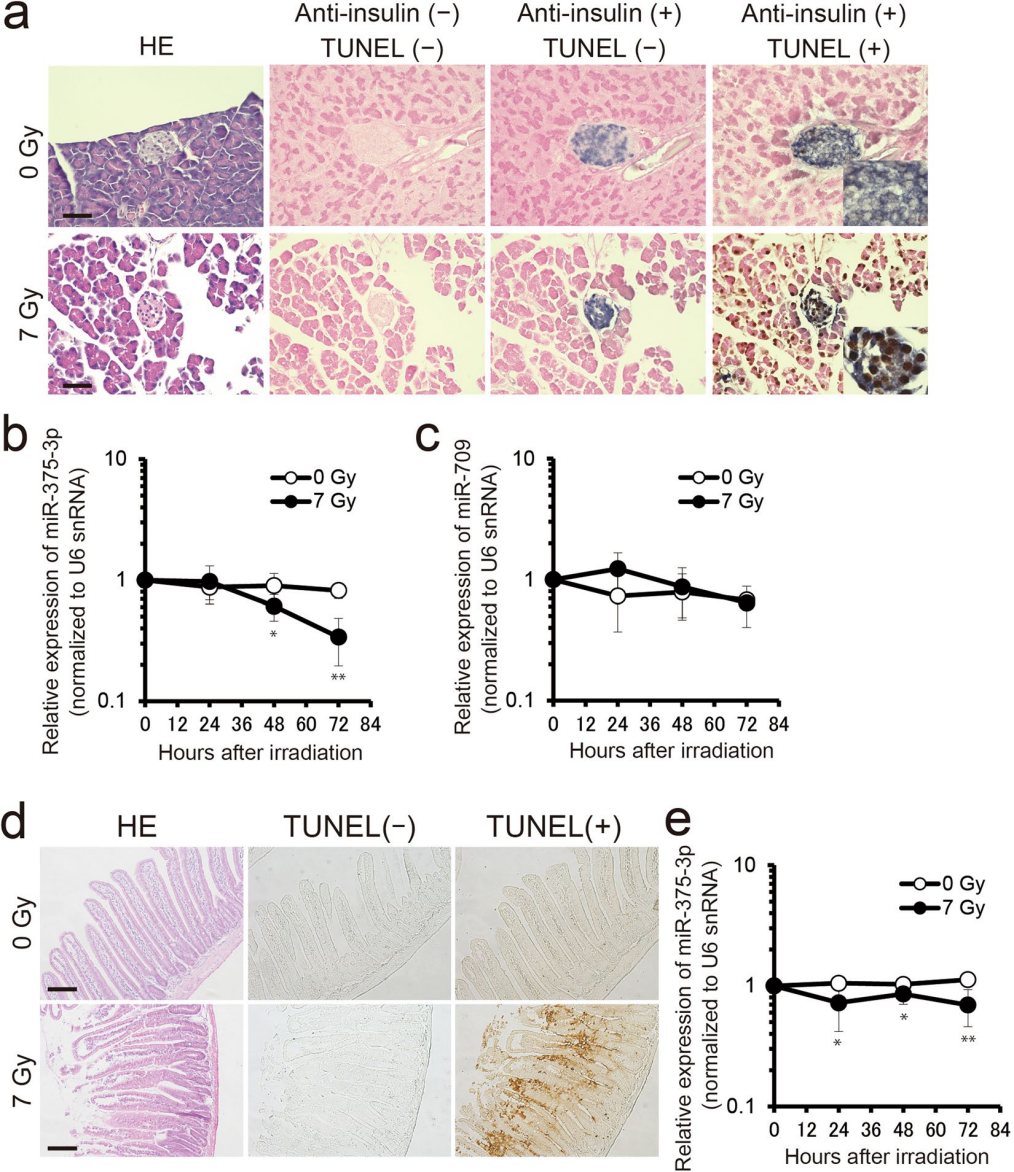
The expression of miR-375-3p decreases in irradiated pancreas and intestine. (**a**) Double staining of terminal deoxynucleotidyl transferase mediated deoxyuridine triphosphate nick end labeling (TUNEL) and immunohistochemistry of insulin in pancreas of control mice and at 72 h after 7 Gy X-irradiation. Hematoxylin and eosin (HE) staining was performed to visualize the fine structures of tissues. Scale bars, 50 μm. (**b** and **c**) The expressions of miR-375-3p and miR-709 measured by RT-qPCR in pancreas at 0 h, 24 h, 48 h and 72 h after 0 Gy and 7 Gy of X-irradiation (each *n* = 6−7). (**d**) TUNEL staining in intestine of mice at 72 h after 7 Gy irradiation. Scale bars, 50 μm. (**e**) The expressions of miR-375-3p in intestine by RT-qPCR at 0 h, 24 h, 48 h and 72 h after 0 Gy and 7 Gy irradiation of X-ray (each *n* = 5−7). U6 snRNA was used as an internal control. Statistical significance was examined by Student’s *t*-test (*P ≤ 0.05, ** *P* ≤ 0.01 vs 0 Gy).

The function of miR-709 in pancreas is not clear, whereas it is reported that miR-375-3p is expressed in pancreatic beta cells and regulates the secretion of insulin^27,28^. In order to analyze whether the release of miRNAs into the blood stream correlates with radiation-induced cell death in pancreatic beta cells, we performed double staining of terminal deoxynucleotidyl transferase mediated deoxyuridine triphosphate nick end labeling (TUNEL) and immunohistochemistry of insulin in mouse pancreas. The results indicated that a dose of 7 Gy induced cell death in pancreatic beta cells (Fig. 5a). In addition, we investigated the expressions of miR-375-3p and miR-709 in pancreas exposed to 7 Gy of X-rays. The expressions of miR-375-3p in pancreas significantly decreased at 48 h and 72 h after 7 Gy irradiation compared with 0 Gy: respectively, 0.68-fold (*P* ≤ 0.05) and 0.41-fold (*P* ≤ 0.01) (Fig. 5b). However, there were no significant differences of the expression of miR-709 in pancreas at 24 h, 48 h and 72 h after 7 Gy irradiation compared with 0 Gy (Fig. 5c).

As shown in Fig. 4a, miR-375-3p is also expressed in the intestine. Therefore, we analyzed cell death and the expressions of miR-375-3p in this organ. Cell death was partially induced in intestine of mice exposed to 7 Gy of X-ray (Fig. 5d). MiR-375-3p in intestine decreased at 24 h, 48 h and 72 h after 7 Gy irradiation as compared with 0 Gy: respectively, 0.69-fold (*P* ≤ 0.05), 0.83-fold (*P* ≤ 0.05) and 0.62-fold (*P* ≤ 0.01) (Fig. 5e). These results suggest that miR-375-3p may leak from cellular to extracellular space due to radiation-induced cell death such as apoptosis and/or necrosis.

### MiR-375-3p is released from pancreatic beta cells exposed to 7 Gy of X-ray to extracellular space *in vitro*

**Figure 6:**
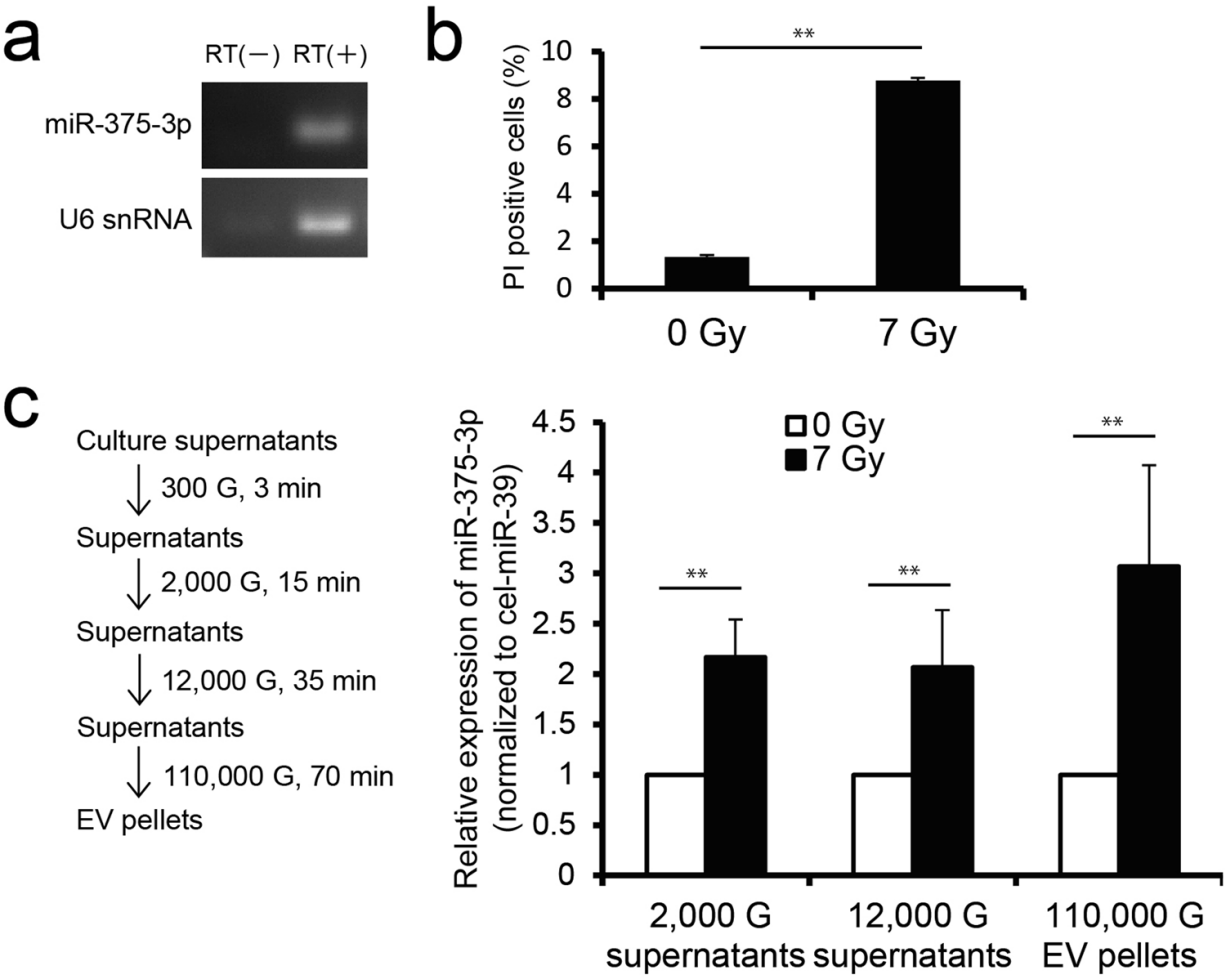
The expression of miR-375-3p in pancreatic beta cell RIN-5F exposed to 7 Gy of X-rays. (**a**) The expression of miR-375-3p and U6 snRNA in pancreatic beta cell RIN-5F by RT-PCR and electrophoresis using 4% agarose gel and ethidium bromide. Negative control was not included in the reverse transcription (RT) reaction. (**b**) Detection of propidium iodide (PI) positive cells by flow cytometry. PI positive cells significantly increased at 72 h after 7 Gy irradiation compared with 0 Gy. (**c**) The expression of miR-375-3p in each centrifuged supernatant and EV sample from RIN-5F cells. Cel-miR-39 was used as external control. Expression levels of miR-375-3p were determined by RT-qPCR using the comparative Ct methods. The data are expressed as means ± standard deviations (each *n* = 4). Statistical significance was examined by Student’s *t*-test (**P ≤ 0.01 vs 0 Gy).

In order to analyze whether pancreatic beta cells release miR-375-3p to extracellular space following high dose radiation exposure, we investigated the expression of miR-375-3p in culture supernatants of pancreatic beta cells RIN-5F exposed to 7 Gy of X-rays. RIN-5F cells expressed miR-375-3p and U6 snRNA (Fig. 6a) indicating that they have the characteristics of pancreatic beta cells. In order to analyze cell death in RIN-5F exposed to 7 Gy of X-rays, propidium iodide (PI) staining and flow cytometry analysis was performed at 72 h p.r. PI positive cells significantly increased at 72 h after 7 Gy irradiation compared with 0 Gy (*P* ≤ 0.01) (Fig. 6b). Next, we investigated whether the released miR-375-3p is free or exists inside EVs in culture medium. We analyzed the expression of miR-375-3p using culture supernatants centrifuged at 12,000 G (including free-miR-375-3p, large EVs, and small EVs), supernatants centrifuged at 12,000 G (including free-miR-375-3p and small EVs), and EV pellets centrifuged at 110,000 G (Fig. 6c). The results demonstrated that miR-375-3p released from RIN-5F cells exposed to 7 Gy of X-rays was included inside EVs. These results suggest that radiation-induced death of pancreatic beta cells is associated with the release of EVs containing miR-375-3p.

## Discussion

The aim of this study was to identify radiation-responsive serum miRNAs in mice exposed to a high dose of X-rays. We found that serum miR-375-3p and miR-709 increased in mice exposed to an X-ray dose of 7 Gy which induced a strong ARS. In addition, we could show that radiation-induced cell death in pancreas and intestine is the major source of the high miR-375-3p level in blood serum. The origin of miR-709 remains unclear.

Our results suggest that radiation-induced injury in the pancreas and intestine is the source of increased miR-375-3p in serum. Therefore, elevated miR-375-3p in serum may be a predictor of tissue damage by exposure to a high radiation dose. This is of particular interest in view of the results from several other groups, who tried to identify miRNAs which are predictive for hematologic ARS. For example, Jacob *et al*. reported that miR-150, abundant in lymphocytes, exhibited a dose and time dependent decrease in mouse serum^25^. Port *et al*. showed that miR-342-3p was the candidate for prediction of hematologic ARS in baboons^29^. Acharya *et al*. reported that miR-30a-3p and miR-30c-5p increased after exposure to a lethal radiation dose^30^. These studies showed differentially expressed serum miRNAs after varying doses of IR which potentially can predict hematologic ARS. However, the mechanisms of their differential expression as well as their origin were not identified. It remains unclear why each study identified a different miRNA as biomarker of exposure. An obvious explanation is that this is due to the use of various species or inbred mouse strains. If this is the reason, then an individual analysis of many people exposed to high radiation doses, such as during the course of radiotherapy, is necessary for identifying appropriate human miRNA biomarkers. Nevertheless, our results and those of others demonstrate the high potential of miRNAs as biomarkers of exposure. The miRNA expression in human cells exposed to a high dose radiation can result in a different cellular response than that induced by a low dose. Chaudhry *et al*. reported that let-7e or *c-Myc* related miRNAs (miR-17-3p, miR-17-5p, miR-19a, and miR-142-5p) were downregulated after 3 h following 100 mGy acute dose in AG1522 normal human skin fibroblasts, but were upregulated after 3 h following 4 Gy acute dose^31^. This indicates that the expression patterns of miRNAs differ after exposure to low and high doses. Consequently, it is possible that miR-375-3p is not a suitable marker of exposure to low radiation doses. This issue requires further analysis because the generally accepted consensus dose for human triage after radiation exposure is 2 Gy.

High-dose IR induces DNA damage and then cell death such as apoptosis or necrosis^32^. Apoptotic cell death is associated with the release of microparticles (apoptotic bodies) which carry miRNAs^33–35^. On the other hand, necrotic cell death leads to the release of intracellular components such as miRNAs to extracellular space. We observed a high level of cell death in pancreatic beta cells and intestine following exposure of mice to 7 Gy of X-rays (Fig. 5a and d). This observation correlated with a significant reduction of the miR-375-3p level in these organs (Fig. 5b and e), which suggests that miR-375-3p may be released from cellular to extracellular space by radiation-induced cell death, leading to its high expression in blood serum (Fig. 6).

A deeper analysis is necessary to identify the relationship between the radiation dose and serum miR-375-3p expression. Analysis of serum miR-375-3p expression as a first screening test of many specimens may be helpful for predicting a strong ARS. The advantage of such test is its speed. It takes over 50 hours from blood sampling to dose assessment by the dicentric and PCC assays^36^, and the processing of blood samples is the bottleneck of these assays when it comes to their use as ‘triage’ tests in large scale radiological emergencies. In our laboratory, it takes about one day (including blood sampling, RNA extraction, synthesis of cDNA and RT-qPCR) for the analysis of serum miR-375-3p. Importantly, the miRNA assay can be further accelerated. For example, changing from a standard RT-qPCR to one-step RT-qPCR performed in one PCR tube and the use of TaqMan MiRNA Cells-to-C_T_ kit (Thermo Fisher Scientific) for quantifying the expression levels of miRNAs directly from lysates may make it possible to reduce the time of analysis to a few hours^37,38^. The development and standardization of suitable tools for diagnosis of miRNA detection in human body fluids is necessary, as reagents for research described above are not used for diagnosis of human disorders.

We found that serum miR-375-3p increased after 48 h and 72 h in mice exposed to an X-ray dose of 7 Gy (Fig. 3a). Biomarkers with an even better time-response may be necessary for quick dosimetric triage in an emergency. Templin *et al*. showed that 45 miRNAs were upregulated 4 h after irradiation with 1.25 Gy X-rays in peripheral blood of radiotherapy patients^39^. However, application of the Paxgene Blood RNA Kit (Qiagen) results in the isolation of total RNA from white blood cells and not the serum. It is known that white blood cell numbers change as a consequence of radiation exposure and this may be a confounding factor in the use of RNA for biodosimetry. Therefore, the identification of novel early miRNA biomarkers for radiation dosimetry in serum is needed.

Using a nanoString nCounter mouse miRNA expression assay (nanoString Technologies), Jacob *et al*. reported that serum miR-375-3p in mice increased already after 24 h following 8 Gy irradiation^25^. 8 Gy is higher than the dose used by us, but it is possible that serum miR-375-3p might serve as an early biomarker of a strong ARS after doses in excess of 7 Gy. It is unclear whether other miRNAs except for miR-375-3p may serve as biomarkers of a strong ARS.

As external control we used the synthesized cel-miR-39 derived from *C*. *elegans* which is not expressed in mammals. Cel-miR-39 was used by a number of researchers^40–42^ for normalization of miRNAs. However, a suitable internal control in body fluids has not yet been discovered. Xiang *et al*. reported that U6 snRNA is not suitable as an internal reference gene in studies of circulating miRNA because the U6 expression gradually decreased after freezing and thawing^43^. It would be desirable to set up an international expert group to discuss the possibility of establishing an international standard for circulating miRNAs.

MiR-375-3p which is highly expressed in pancreatic beta cells regulates the expression of pyruvate dehydrogenase kinase, isozyme 1 (*Pdk1*) and myotrophin (*Mtpn*) mRNAs^44,45^. Moreover, it regulates the development of beta cells and the secretion of insulin by up-/down regulation of *Pdk1* and *Mtpn* mRNAs^28,46,47^. It was reported that miR-375-3p increases in mouse plasma of type I diabetes model mice treated by streptozotocin which specifically injures pancreatic beta cells^48^. It indicates that injury of pancreatic beta cells is the major source of increased miR-375-3p levels in the blood. If point-of-care testing of serum/plasma miR-375-3p as a biomarker of type I diabetes develops, this system may be able to be used as a biomarker of a lethal dose received by victims of severe radiation accidents.

## Methods

### Ethic statement

All experiments were performed in accordance with guidelines for Animal Experimentation of Hirosaki University, and the procedures were approved and monitored by the Animal Research Committee of Hirosaki University (approved number: G12003).

### Mice and X-ray irradiation

Male C57BL/6NJcl mice were delivered at 7 weeks of age from the breeding facilities of Clea, Japan. 8 weeks old mice were exposed to X-rays (MBR-1520R-3 X-ray machine, Hitachi Medical Corporation, Tokyo, Japan) at a dose rate of 1.0 Gy/min (150 kVp, 20 mA, 0.5 mm aluminum and 0.3 mm copper filters). The irradiated and non-irradiated mice were sacrificed after 0 h, 24 h, 48 h, and 72 h p.r. for collection of blood and organ tissues. The numbers of mice used for each experiments are indicated in figure legends. Sera were obtained from blood using BD Microtainer tubes with serum separator additive BD Microgard closure (Becton Dickinson Biosciences, Rockville, MD, USA). EVs in serum were collected using an ExoQuick Solution (System Biosciences). The number of PWBCs and bone marrow cells were counted using the Burker-Tulk solution and a counting chamber.

For tissue analyses, tissues from mice were first fixed *in situ* by perfusion with 4% (w/v) ice-cold paraformaldehyde solution (PFA) in Dulbecco’s phosphate-buffered saline (D-PBS, pH7.2). The resulting tissues were excised and further fixed overnight in the 4% PFA solution. Bones were demineralized for 1 week using a 10% (w/v) ethylenediaminetetraacetate acid solution. The fixed tissues were embedded in paraffin and 4 µm sections were cut. Sections were placed on glass slides and subjected to hematoxylin-eosin staining, TUNEL method, and/or immunohistochemistry staining. All mice were housed in a conventional animal room with 12 hours light/dark cycles, with food and water accessible *ad libitum*.

### RNA extraction

RNAs from serum and cells/tissues were extracted using the Isogen II reagent and ethachinmate (Nippongene) according to manufacturer’s instruction. The concentration of extracted serum RNAs were examined using a Quant-iT RiboGreen RNA Reagent and kit (Thermo Fisher Scientific) and a Fluoroskan Ascent (Thermo Fisher Scientific) according to manufacturer’s instructions. In addition, the peaks of small RNAs were confirmed using Agilent 2100 Bioanalyzer and Agilent RNA 6000 Pico kit (Agilent Technologies), according to manufacturer’s instructions. The quality and concentration of cells/tissues RNAs were assessed using the NanoDrop spectrophotometer (NanoDrop Technologies, Wilmington, DE, USA). All cell/tissue RNA samples had 260/280 nm absorbance ratios of 1.8–2.0.

### Microarray analysis

Cy3-labeled miRNA was synthesized from 30 ng total RNA of irradiated (7 Gy) and non-irradiated (0 Gy) sera using a miRNA Complete Labeling Reagent and Hyb kit (Agilent Technologies). A SurePrint G3 mouse miRNA microarray slide (8 × 60 K, Ver.21.0) was hybridized with the Cy3-labeled miRNA in a hybridization solution prepared with a Gene Expression Hybridization Kit (Agilent Technologies), following the manufacturer’s instructions. Cy3 fluorescence signal images on the slide were obtained by a SureScan microarray scanner (Agilent Technologies), and processed using the Feature Extraction version 10.7 software based on the instructions from Agilent Technologies. The expression data thus obtained were processed using GeneSpring GX14.5 software (Agilent Technologies) in order to make a normalization to percentile shift 90% of all values on the respective microarrays, followed by normalization of the median expression level of all samples. The expression profiles of miRNAs were compared, based on the fold-change of the signal values (Lower cut-off; 100) of respective genes and Student’s *t*-test. The increasing and decreasing miRNAs of more than 1.5-fold at 7 Gy against 0 Gy were selected. Results are visualized with the help of heat maps and dendrograms. The heat maps show color-coded expression levels; the color gradation from red to green indicates the expression levels from high to low. Sample trees were drawn horizontally and gene trees were drawn vertically.

### RT-qPCR

Expression levels of miRNAs from sera and/or tissues were analyzed using cDNAs that were synthesized from miRNAs using TaqMan miRNA RT kits and the prescribed 5 × RT primer (both from Thermo Fisher Scientific) according to the manufacturer’s instructions. Subsequently, qPCR for miRNAs was performed using FastStart TaqMan probe master (Roche Diagnostics, Basel, Switzerland), a 20 × probe, and a StepOne Plus real-time PCR system (Thermo Fisher Scientific) under the following conditions: 10 min at 95 °C, followed by 40 cycles at 95 °C for 15 sec and 60 °C for 60 sec. RNAs were isolated from 50 or 200 μl aliquots of sera following the addition of 5 μl of 1 nM cel-miR-39. Cel-miR-39 was used as an external control and U6 small nuclear RNA (snRNA) was used as an internal control. Expression levels were determined using the comparative Ct method. The PCR products were detected by electrophoresis using 4% agarose gel and ethidium bromide.

### TUNEL method

Paraffin-embedded sections of pancreas and intestine isolated from mice irradiated by 7 Gy of X-ray on glass slides were deparrafinized with xylene and ethanol, washed in PBS and treated with 0.3% hydrogen peroxide for 10 min. The slides were washed in PBS solution and fixed in 4% PFA solution for 15 min. The slides were treated with 20 µg/ml proteinase K solutions for 30 min at room temperature, washed in PBS solution for 5 min each time and fixed in 4% PFA solution for 5 min. To block endogenous biotin, avidin solution (Agilent Technologies) was treated for 10 min, biotin solution (Agilent Technologies) then was treated for 10 min and washed in PBS. The cell death assay was performed using a DeadEnd Colorimetric TUNEL System (Promega, Madison, WI, USA). The slides were incubated in equilibration buffer for 10 min. To label biotinyl nucleotides in 3’-OH of DNA nick, biotinyl nucleotides and terminal deoxynucleotidyl transferase solution were treated with slides for 60 min at 37 °C. Then, the slides were washed in 2 × saline sodium citrate buffer for 15 min and PBS for 5 min. Horseradish peroxidase labeled streptavidin diluted in PBS (1:500) was dropped on the slides and incubated for 30 min. The slides were washed in PBST (PBS in 0.1% tween 20). Signals were visualized using 3,3-diaminobenzidine (Sigma-aldrich, St. Louis, MO, USA).

### Immunohistochemistry

Immunohistochemistry staining was performed for detection of insulin in pancreatic beta cells. TUNEL-stained slides were washed in water and treated with 0.2 N HCl for 15 min. The slides were treated with 10 mM citrate buffer (pH 6.0) and incubated for 10 min at 121 °C using an autoclave. Then, the slides were washed in TBS buffer (25 mM Tris-HCl and 150 mM NaCl, pH 7.2). To perform blocking, the slides were treated with the blocking solution (5% normal goat serum in TBS buffer) at room temperature for 20 min. Next, the slides were incubated with the blocking solution containing a primary rabbit monoclonal antibody directed against mouse insulin (#3014, Cell Signaling Technology, Danvers, MA, USA) at a 1:2000 dilution at room temperature for 60 min. The slides were washed three times with TBS buffer and incubated at room temperature for 60 min with an goat anti-rabbit IgG alkaline phosphatase-conjugated secondary antibody (#7054, Cell Signaling Technology) prepared in the blocking solution at a 1:5000 dilution. The slides were washed twice with NT buffer (150 mM NaCl and 100 mM Tris-HCl, pH 7.5) for 5 min and once with NTM buffer (100 mM NaCl, 50 mM MgCl_2_ and 100 mM Tris-HCl, pH 9.5) for 5 min. Signals were detected with the nitro blue tetrazolium chloride/5-bromo-4-chloro-3-indolyl-phosphate (NBT/BCIP) working solution [NBT/BCIP stock solution (Sigma-aldrich) prepared in the NTM buffer with a 1:50 dilution]. The slides were washed in water and background was stained using an eosin solution. Finally, the slides were dehydrated with ethanol and xylene, and mounted using a hydrophobic mounting agent.

### Cell line and culture

The pancreatic beta cell RIN-5F was purchased from the American Type Culture Collection (ATCC, Manassas, VA, USA). RIN-5F cells were cultured in RPMI-1640 medium (Wako, Tokyo, Japan) supplemented with 10% fetal bovine serum (Thermo Fisher Scientific), 100 U/ml of penicillin, and 100 µg/ml of streptomycin (Wako). Cells were cultured at 37 °C in a humidified atmosphere with 5% CO_2_.

### Flow cytometry

RIN-5F cells were plated on 10-cm dishes at a density of 1 × 10^6^ cells per dish in the culture medium. After 96 h, culture medium was discarded, cells were washed three times in D-PBS, and 10 ml culture medium was added to each dish. RIN-5F cells were then exposed to 7 Gy of X-rays at a dose rate of 1.0 Gy/min. After 72 h, the medium was used for purification of EVs as described below. The irradiated RIN-5F cells were collected and stained by PI solution. PI positive cells were detected using a Cytomics FC500 (Beckman Coulter, Brea, CA, USA).

### Purification of EVs from culture medium

Cell culture medium samples described above were centrifuged at 300 × G at 4 °C for 3 min to remove floating cells. The supernatants were then centrifuged at 2,000 × G at 4 °C for 15 min, and RNAs were extracted from 200 µl of the collected supernatants using Isogen II reagents. Furthermore, the collected supernatants were centrifuged at 12,000 × G at 4 °C for 35 min, and RNAs were extracted in the same way. Finally, the supernatants were ultracentrifuged at 110,000 × G at 4 °C for 70 min in an Optima TLX Ultracentrifuge (Beckman Coulter) to collect EV pellets.

### Statistical analysis

Statistical analysis was performed using Excel 2010 (Microsoft, Redmond, WI, USA) with the add-in software Statcel 3 (OMS publishing Inc., Saitama, Japan) and the Origin software package (OriginLab Pro v8.1; Northampton, MA, USA). Survival differences were examined by the log-rank test. Statistical significance (*P* ≤ 0.05) was examined by Tukey-Kramer test for multiple comparisons. Student’s *t*-test was used to compare the results of two groups.

## Electronic supplementary material

Supplementary Information
