# Supplementary material for: Serum miR-375-3p increase in mice exposed to a high dose of ionizing radiation
Source: Sci Rep. 2018 Jan 22;8:1302. doi: 10.1038/s41598-018-19763-7 (PMC5778023; doi:10.1038/s41598-018-19763-7)
Supplement: Supplementary file 1 — Supplementary Information [file 41598_2018_19763_MOESM1_ESM.pdf]

Supplementary Information

Serum miR-375-3p increase in mice exposed to a high dose of ionizing radiation

Mitsuru Chiba[1,*], Satoru Monzen[2], Chihiro Iwaya[3], Yuri Kashiwagi[3], Sunao Yamada[3], Yoichiro Hosokawa[2], Yasushi Mariya[4], Toshiya Nakamura[1], and Andrzej Wojcik[5,6]

[1] Department of Bioscience and Laboratory Medicine, Graduate School of Health Sciences, Hirosaki University, 66−1, Hon-cho, Hirosaki, Aomori 036-8564, Japan

[2] Department of Radiation Sciences, Graduate School of Health Sciences, Hirosaki University, 66−1, Hon-cho, Hirosaki, Aomori 036-8564, Japan

[3] Department of Medical Technology, Hirosaki University School of Health Sciences, 66−1, Hon-cho, Hirosaki, Aomori 036-8564, Japan

[4] Department of Radiology and Radiation Oncology, Mutsu General Hospital, 1−2−8, Kogawa-machi, Mutsu, Aomori 035-0071, Japan

[5] Department of Molecular Biosciences, The Wenner Gren Instititute, Stockholm University, Svante Arrhenius väg 20C, 10691 Stockholm, Sweden

[6] Department of Radiobiology and Immunology, Institute of Biology, Jan Kochanowski University, ul. Swietokrzyska 15, 25-406 Kielce, Poland

*Corresponding author: Mitsuru Chiba, Ph.D.
Department of Bioscience and Laboratory Medicine, Graduate School of Health Sciences, Hirosaki University, 66−1, Hon-cho, Hirosaki, Aomori 036-8564, Japan
E-mail: mchiba32@hirosaki-u.ac.jp

Supplementary Table S1. Expression of increasing 12 miRNAs and decreasing 6 miRNAs in serum of mice exposed to 7 Gy of X-rays.

miRNA name	Fold changes	Regulation	miRBase accession numbers
mmu-miR-5126	3.11692	Up	MIMAT0020637
mmu-miR-8110	3.18392	Up	MIMAT0031416
mmu-miR-3473b	1.81603	Up	MIMAT0020367
mmu-miR-5100	2.29878	Up	MIMAT0020607
mmu-miR-709	2.93291	Up	MIMAT0003499
mmu-miR-3470a	2.43230	Up	MIMAT0015640
mmu-miR-375-3p	2.49345	Up	MIMAT0000739
mmu-miR-3473f	3.04175	Up	MIMAT0031390
mmu-miR-1897-5p	4.79886	Up	MIMAT0007864
mmu-miR-5128	3.45978	Up	MIMAT0020639
mmu-miR-7082-5p	2.50765	Up	MIMAT0028070
mmu-miR-7118-5p	2.63865	Up	MIMAT0028133
mmu-miR-29a-3p	-4.27230	Down	MIMAT0000535
mmu-miR-140-3p	-6.55231	Down	MIMAT0000152
mmu-miR-92a-3p	-7.76988	Down	MIMAT0000539
mmu-miR-30e-5p	-2.77734	Down	MIMAT0000248
mmu-miR-222-3p	-3.55784	Down	MIMAT0000670
mmu-miR-425-5p	-3.13122	Down	MIMAT0004750
